# Defining the role of 2,2’,4,4’-tetrabromodiphenyl ether in 3T3-L1 cellular differentiation by transcriptome sequencing analysis

**DOI:** 10.1080/21623945.2024.2430717

**Published:** 2024-12-07

**Authors:** Zao-Ling Liu, Aerna Qiayimaerdan, Yong Fan, Shu-Rui Jiang, Zunire Tuerxuna, Meng-Lin Wang, Haiqiemuhan Abudureheman

**Affiliations:** a Department of Epidemiology & Health Statistics, School of public health, Xinjiang Medical University, Urumqi, Xinjiang, China; b Department of Endocrinology, The First Affiliated Hospital of Xinjiang Medical University, Urumqi, China

**Keywords:** 3T3-L1, BDE-47, adipogenesis, transcriptome sequencing, mitosis

## Abstract

This study aims to investigates the effect of 2,2’,4,4’-tetrabromodiphenyl ether (BDE-47) on the differentiation of 3T3-L1 cells and its mechanism of action. These 3T3-L1 cells were induced to differentiate in vitro using methylisobutylxanthine, dexamethasone, and insulin conditions, then exposed to either 1% DMSO as a control group or varying concentrations of BDE-47 (2.5 μM, 7.5 μM, 12.5 μM, 18.75 μM, and 25 μM). Oil red O staining showed that the absorbance value of the BDE-47 exposure groups was higher than that of the control group (*p* < 0.05). This study identified 722 common genes between the differentially expressed genes of each exposure group. Using Cytoscape 10 hub genes were identified as Actb, Cdk1, Myc, Ccnb1, Aurkb, Plk1, Aurka, Pparg, Kif11, and Casp3. Enrichment analysis data revealed that the effects of BDE-47 on 3T3-L1 cell differentiation were associated with the cell cycle, p53 signalling, and PPARγ pathways. The transcription factor genes, KAT2A, MAX, SIN3A, TBP, and EP300, were shown to be associated with the PPARγ pathway. The mRNA expression of PPARγ in each exposure group was higher than that in the control group (*p* < 0.05), and a bimodal distribution between PPARγ mRNA expression and BDE-47 dose was observed. These findings indicate that BDE-47 May activate the PPARγ pathway and mitotic pathway to regulate the cell cycle and induce adipocyte differentiation.

## 1. Introduction

Obesity is a global epidemic disease and a risk factor for heart metabolic complications, such as insulin sensitivity, type 2 diabetes, and non-alcoholic fatty liver disease [1]. At the cellular level, obesity is the result of the abnormal differentiation of adipocytes, including an increase in the number of adipocytes and an increase in the volume of a single adipocyte [2]. Adipogenesis is the process where fibroblast-like progenitor cells become part of the adipogenic lineage, accumulate nutrients and become triglyceride-filled mature adipocytes [3]. The normal differentiation process of adipocytes is: multifunctional stem cells, adipoblasts, preadipocytes, and mature adipocytes [4]. Preadipocytes are regulated by a variety of transcription factors. Many related genes are activated during cell differentiation, and then preadipocytes eventually differentiate into mature adipocytes under the coordination of these genes [5]. Abnormal adipocyte differentiation and transcriptional regulation not only leads to abnormally elevated insulin levels, but also produces insulin resistance, which is closely correlated with the development of various metabolic diseases such as obesity, diabetes, and non-alcoholic fatty liver disease [6]. Adipogenesis is a complex process accompanied by changes in morphology, hormones, and gene expression [7].

At present, obesity is generally considered to be affected by both genetic and environmental factors [8]. Endocrine disruptors, such as bisphenol A (BPA), polycyclic aromatic hydrocarbons (PAHs), and polybrominated diphenyl ethers (PBDEs), are chemicals commonly found in the environment that can interfere with the endocrine system [9]. These PBDEs are common brominated flame retardants, and due to their long-distance migratory ability in the environment, high lipophilicity, and persistence, they are present in both the human body and environment [10]. In recent years, studies have shown that BDE-47 May also be an environmental risk factor for metabolic diseases such as obesity, insulin resistance, type 2 diabetes, and hypertension [11,12]. At the molecular level, BDE-47 May abnormally regulate the terminal differentiation of preadipocytes, promote adipogenesis, and cause abnormal lipid metabolism, further affecting the role and function of metabolic disease-related factors, and leading to the occurrence and development of diseases [13,14]. However, the specific mechanism of BDE-47 affecting preadipocyte differentiation needs to be further studied. This study selected BDE-47 as the test substance due to its high content in the environment, strong biological toxicity, wide distribution, and high detection rate in animals and humans [15].

The 3T3-L1 cells are mouse embryonic fibroblasts, also known as preadipocytes [16]. Under appropriate induction conditions, 3T3-L1 cells can differentiate into mature adipocytes in vitro, through a process known as lipogenic induction [17] and are currently used in studies examining the treatment of obesity, diabetes, and other diseases. Studying the process of adipocyte proliferation and differentiation is of great significance to understand the occurrence of obesity, as well as the prevention and treatment of related diseases [18]. Previous investigations have delved into the effects of 2,2‘,4,4’-tetrabromodiphenyl ether (BDE-47) on the differentiation of adipocytes, suggesting its role as an environmental factor contributing to obesity (Liu et al., 2022). Despite these findings, a comprehensive understanding of the mechanisms through which BDE-47 exerts its influence has yet to be fully established. Our study seeks to build upon this foundational research by examining the intricate interactions between BDE-47 and key regulatory components of the PPARγ signalling pathway, as well as its impact on cell cycle progression. By doing so, we aim to provide novel insights into the molecular underpinnings of BDE-47’s role in adipocyte development and its potential implications for metabolic health [19]. Therefore, in the present study, 3T3-L1 cells were exposed to different concentrations of BDE-47 to determine the effect of BDE-47 exposure on preadipocyte differentiation. The mechanism of action of BDE-47 exposure on 3T3-L1 cell differentiation was further examined using bioinformatics methods.

The aim of the present study was to utilize various concentrations of BDE-47 to stimulate 3T3-L1 cells, assessing the impact and mechanism of BDE-47 on adipocyte differentiation. This involved investigating the mechanism of BDE-47 exposure on 3T3-L1 cell differentiation through screening hub genes and enriching pathway analysis of transcriptome sequencing.

## 2. Results

### 2.1. Oil red O staining analysis

**Figure 1.**
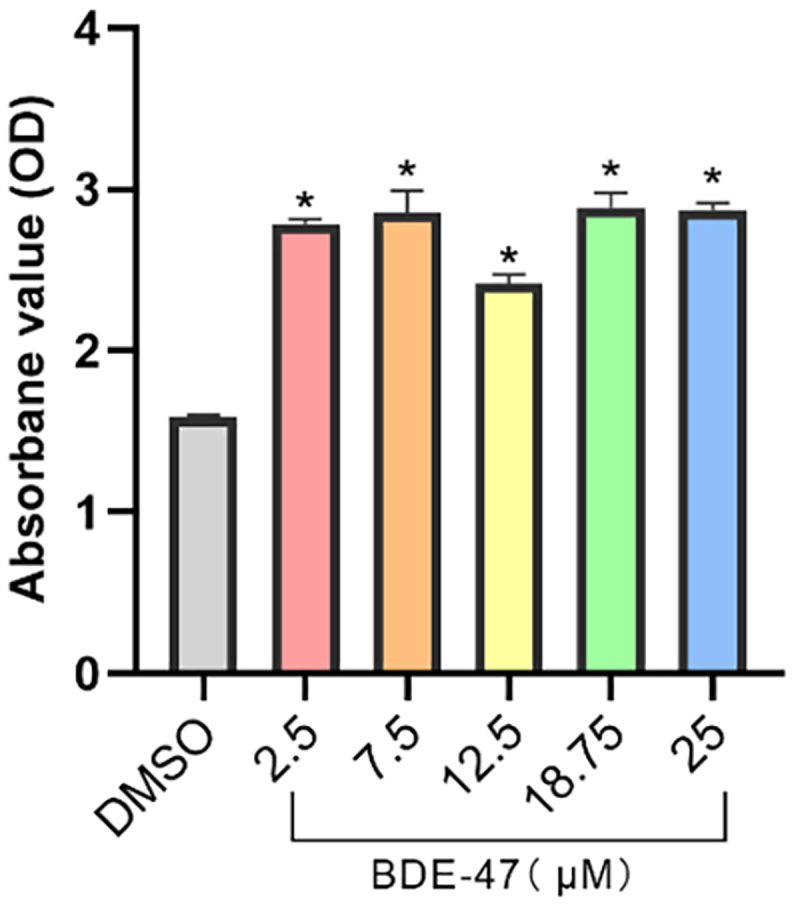
Absorbance value (OD) of oil red O staining for each BDE-47 treatment group.

The absorbance value of oil red O staining on day 8 of cell differentiation was measured to indirectly assess the degree of 3T3-L1 differentiation and it was found that the absorbance values of each BDE-47 group were higher than those of the control group (*p* < 0.05).The absorbance value of BDE-47 treatment groups displayed a bimodal distribution with the lowest absorbance value observed at 12.5 μM BDE-47, and higher absorbance values at the lowest (2.5 μM) and highest (25 μM) BDE-47 concentrations (Figure 1).

### 2.2. Identification of DEGs and common genes

**Figure 2.**
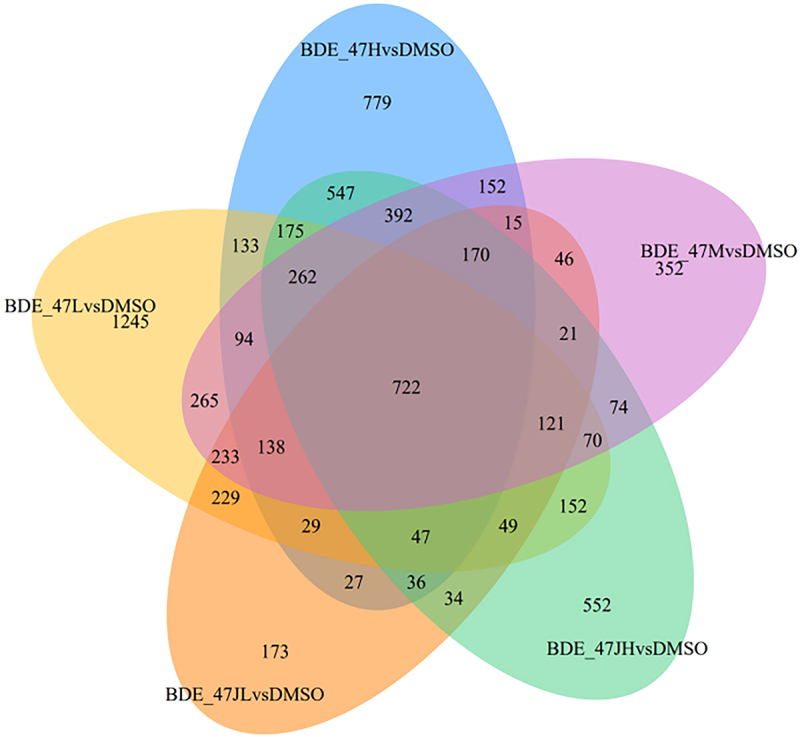
The most common differentially expressed genes in the BDE-47 treatment groups.

Differences in gene expression between the BDE-47-exposure groups and the control group were analysed using the R 4.0.1 limma package. Compared with the control group, 2090 DEGs were obtained including 1022 upregulated and 1068 downregulated DEGs in the BDE-47JL (2.5 μM) group, 3964 DEGs including 1855 upregulated and 2109 downregulated DEGs in the BDE-47 L (7.5 μM) group, 3127 DEGs including 1568 upregulated and 1559 downregulated DEGs in the BDE-47 M (12.5 μM) group, 3718 DEGs including 1853 upregulated and 1865 downregulated DEGs in the BDE-47 h (18.75 μM) group, and 3424 DEGs including 1651 upregulated and 1773 downregulated DEGs in the BDE-47JH (25 μM) group. Figure 2 utilizes a Venn diagram to illustrate the intersections of the most common differentially expressed genes (DEGs) across five BDE-47 datasets. The diagram identifies a total of 722 overlapping genes, which were subsequently selected for further analysis.

### 2.3. Short time-series expression Miner

**Figure 3.**
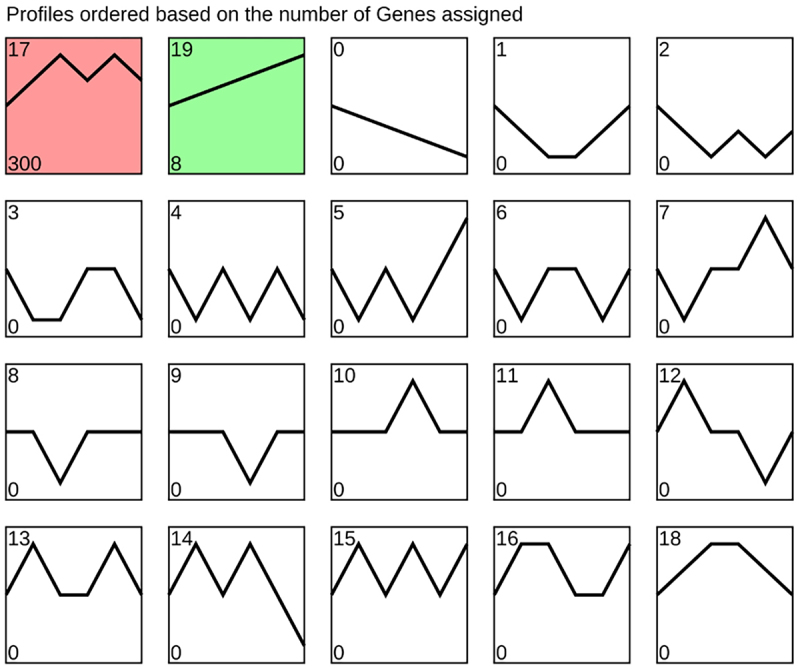
The STEM Analysis of 722 DEGs.

Using STEM analysis,722 common genes were analysed and two trends obtained in Figure 3 (*p* < 0.05). The profile 17 pattern contains 300 genes, and the modal showed the characteristics of first increasing, then decreasing, and then increasing with the increase of BDE-47 dose, which is like the relationship between adipocyte differentiation and BDE-47 concentration. The profile 19 pattern contains 8 genes, which is characterized by up regulation with the increase of BDE-47 dose.

### 2.4. Identification of hub genes

The 722 common genes were imported into the Cytoscape software and the cytoHubba plug-in was used to identify hub genes. The top 10 hub genes identified by the degree algorithm are shown in Table 3 and include *Actb*, *Cdk1*, *Myc*, *Ccnb1*, *Aurkb*, *Plk1*, *Aurka*, *Pparg*, *Kif11*, and *Casp3*. Of these hub genes, *Cdk1*, *Ccnb1*, *Aurkb*, *Plk1*, *Aurka*, and *Kif11* are closely related to the cell cycle and mitosis.

### 2.5. Gene set enrichment analysis

**Figure 4.**
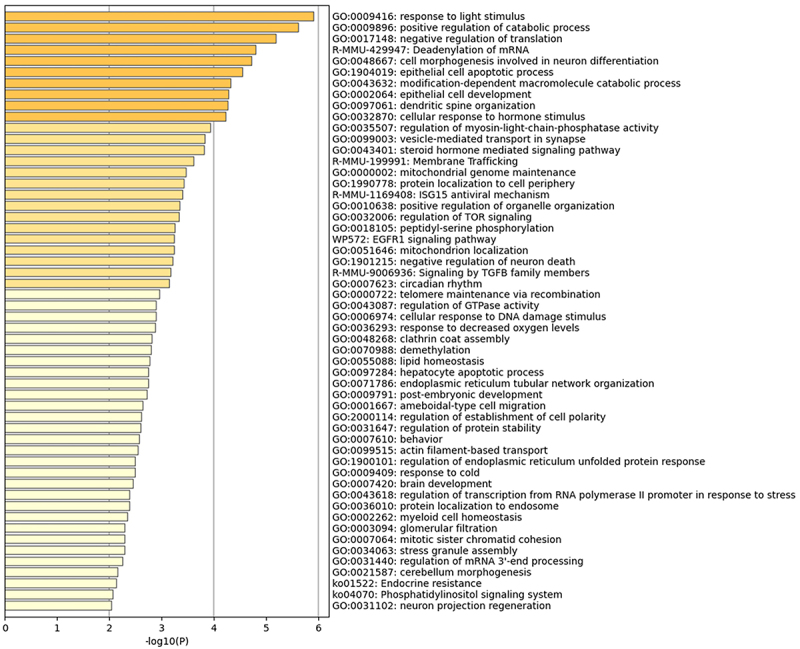
The enrichment analysis of 300 genes from the profile 17.

The current study analysed GO terms, KEGG pathway, Reactome pathway, and WikiPathway for 300 genes in profile 17 and 10 hub genes. Three GO terms were identified including biological process, molecular function, and cellular component. The top 5 enrichment terms of profile trend were: response to light stimulus, positive regulation of catabolic process, negative regulation of translation, deadenylation of mRNA, and cell morphogenesis involved in neuron differentiation (Figure 4). The top 10 GO terms of hub genes for each of the subsections are shown in Table 4. The GO enrichment analysis revealed that the hub genes were mostly enriched in pathways related to the cell cycle and mitosis. The KEGG (ID: mmu04115), Reactome (MMU-6804114), and WikiPathways (WP2902) all indicated that the hub genes *Cdk1*, Ccnb1, *Casp3*, and *Aurka* were enriched in the p53 signalling pathway. The reactome pathway analysis showed that the APC/C-related pathway (MMU-174143 and MMU-176412) and the *Pparg* gene transcription pathway of MMU-74160 and MMU-212436 interacted with the largest number of hub genes (Table 5). The PPARγ pathway was associated with the following genes: *Pparg*, *Cdk1*, *Aurkb*, *Myc*, *Aurka*, *Ccnb1*, and *Actb*.

### 2.6. Transcription factor genes and hub genes interactions

**Figure 5.**
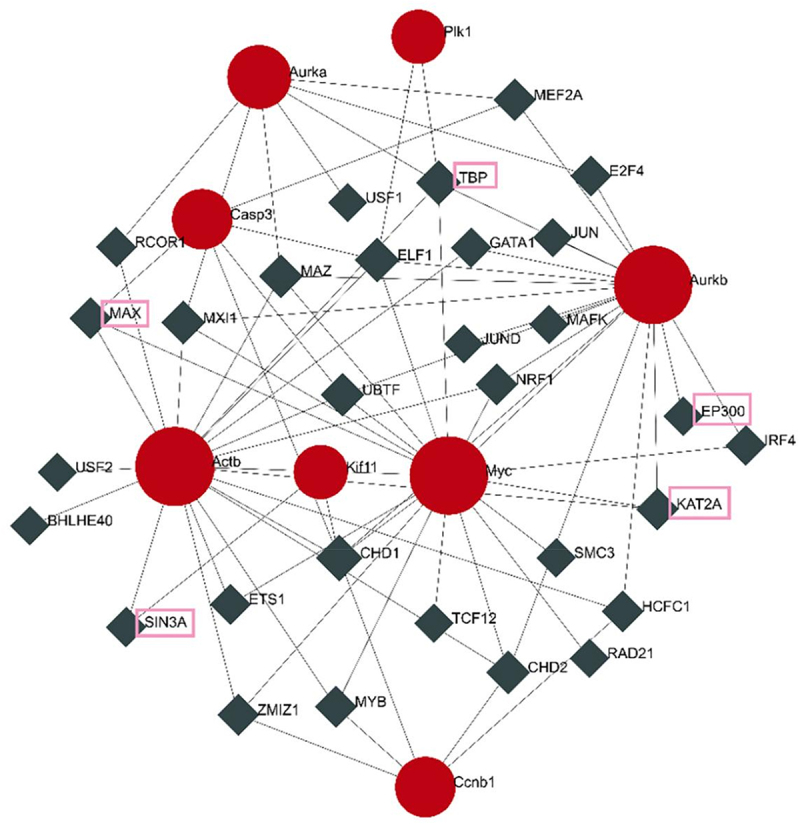
Interactions between transcription factor genes and hub genes.

**Figure 6.**
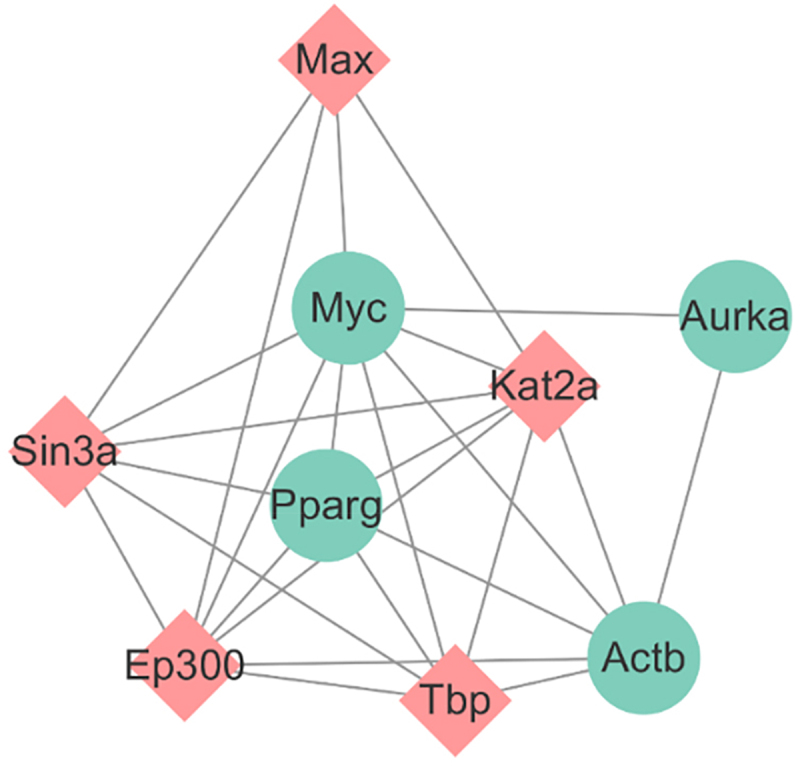
The Pparg transcription interaction network.

The transcription factor genes and hub genes interaction network were established using the NetworkAnaylst database. As shown in Figure 5 The network consisted of 38 nodes and 77 edges. The GeneCards database was used to query the PPARγ pathway (ID: MMU-74160) and found that the transcription factor genes that interacted with hub genes in this pathway were *KAT2A*, *MAX*, *SIN3A*, *TBP*, and *EP300* (Figure 5, genes in pink rectangles). The common hub genes in the PPARγ pathway included *Aurkb*, *Myc*, *Actb*, and *Aurka*. The transcription factor genes and hub genes that appeared in the PPARγ pathway were integrated. As shown in Figure 6, the interaction network consisted of 5 hub genes and 5 transcription factor genes, with 10 nodes and 29 edges.

### 2.7. The expression of PPARγ mRNA

**Figure 7.**
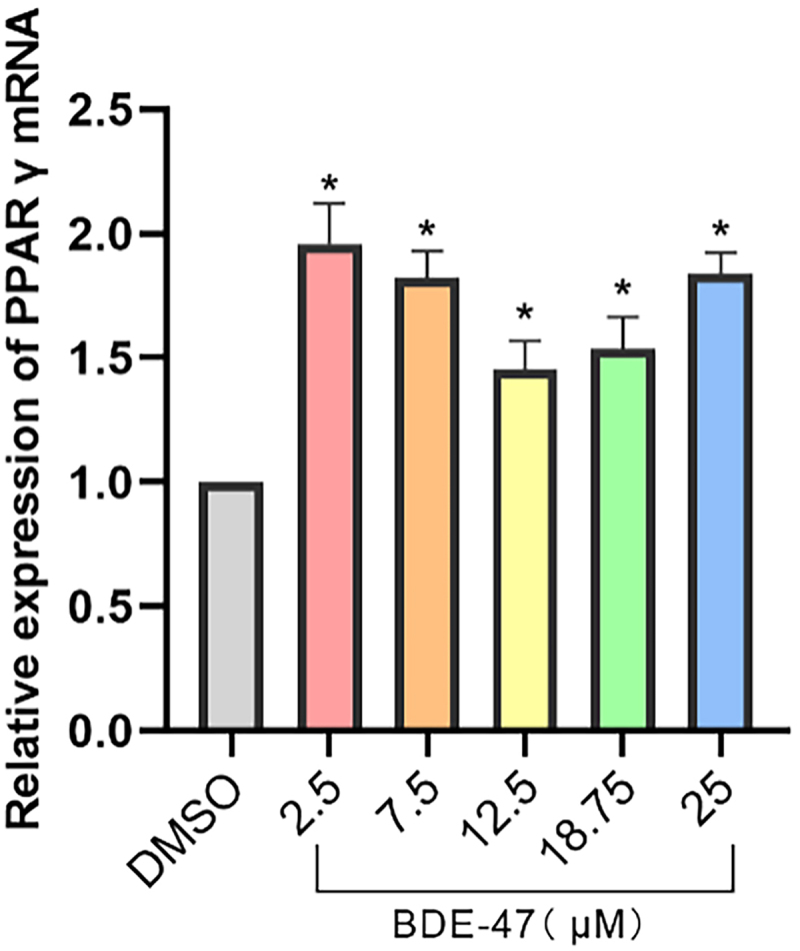
The effects of exposure to different concentrations of BDE-47 on PPARγ mRNA expression levels.

qPCR analysis indicated that the expression of PPARγ in 7.5 μM BDE-47, 12.5 μM BDE-47, 18.75 μM BDE-47, and 25 μM BDE-47 treatment groups was significantly up-regulated compared to the DMSO control group (*p* < 0.05). The mRNA expression of PPARγ displayed bimodal distribution, with the lowest PPARγ mRNA levels observed at 12.5 μΜ BDE-47, and the highest expression found at the lowest (2.5 μΜ) and highest (25 μΜ) doses of BDE-47. This bimodal distribution trend was consistent with the oil red O staining data (Figure 7).

## 3. Discussion

The term ‘environmental obesity factor’ is generally considered to describe a chemical substance that promotes obesity, and covers many environmental pollutants that have been identified as inducing obesity [20]. BDE-47 is one of the most abundant PBDE homologues detected in the human body [21]. This study, exposed 3T3-L1 cells to five different doses of BDE-47 to observe the effect of BDE-47 on adipocyte differentiation. The results showed that BDE-47 significantly promoted the number of 3T3-L1 cells differentiating into adipocytes, indicating that BDE-47 has a specific role in promoting adipocyte differentiation, with exposure causing differential expression of adipocyte differentiation-related genes. Enrichment analysis showed that BDE-47 May activate the PPARγ pathway and mitotic pathway, which regulate the cell cycle and promote adipocyte differentiation.

The effects of exposing 3T3-L1 cells to different concentrations of BDE-47 on 3T3-L1 differentiation were examined. The degree of differentiation was assessed by staining 3T3-L1 cells with oil red O on day 8 of differentiation. The data revealed that compared with the control groups, the number of adipocytes in the BDE-47-exposed group was significantly increased, and that the absorbance value in the medium dose group was significantly lower than that in other groups. Tung et al. [22] induced 3T3-L1 cells with BDE-71 and BDE-47 without DEX, and found that compared with the control group, the degree of 3T3-L1 differentiation induced by 12 μM BDE-47 was increased by approximately 1.5 times, which is like these results. Yang et al. [23] found that treatment of BDE-47 at 5 or 10 μM increased the amount of lipid droplets in differentiated 3T3-L1 cells. However, no further increase was detected in adipocytes when treated with 20 μM BDE-47, which significantly suppressed 3 T3-L1 cell proliferation as measured. On the contrary, 12.5 μM BDE-47 induced 3T3-L1 cells to the lowest extent, while 2.5 and 25 μM induced the highest degree in this study. Bastors et al. [24] showed that the differentiation increases of 3T3-L1 cells was concentration-dependent in 2.5–25 μM BDE-47, while 2.5 μM BDE-47 barely didn’t work. Similarly, in the study of Tung et al., 1.5 μM BDE-47 did not significantly induce adipocyte differentiation [22]. But in our study, the significant increase of adipocyte differentiation was also observed in 2.5 μM BDE-47. In addition, Qing et al. [25] induced 3T3-L1 cells using different doses of BDE-99 (0.01-30 μM) and found a significant increase in adipocyte differentiation. A bimodal distribution was observed between the degree of adipocyte differentiation and BDE-47 exposure dose, suggesting that more than one point of action for BDE-47 which all concentration-dependent. To our knowledge, this is the first time that this phenomenon has been reported. In general, the adipogenic and obesogenic effects of BDE-47 have been well verified, but reports showed that the action concentration and characteristics of BDE-47 are different, and the specific reasons need to be further studied.

Next, this study we screened 722 common DEGs found in each BDE-47 exposure group. Gene expression trend analysis obtained two trends related to the concentration gradient of BDE-47. Among them, the expression characteristics of profile 17 genes were like the differentiation of 3T3-L1 cells. The using of Cytoscape identified *Actb*, *Cdk1*, *Myc*, *Ccnb1*, *Aurkb*, *Plk1*, *Aurka*, *Pparg*, *Kif11*, and *Casp3* as the top 10 hub genes, which were then subjected to gene set enrichment analysis. It was found that the hub genes were associated with mitosis, cell cycle, p53 signalling, APC/C, G2/M, and Pparg-related gene transcription pathways. Of the identified hub genes, *Cdk1*, *Ccnb1*, *Aurkb*, *Plk1*, *Aurka*, and *Kif11* are associated with the cell cycle and mitosis. Furthermore, *Pparg*, a peroxisome that controls the fatty acid β-oxidative pathway, is a key regulator of adipocyte differentiation [26]. In previous studies, Kamstra et al. [27] proposed that BDE-47 May affect PPARγ2 induce cell differentiation. BDE-99 induces 3T3-L1 cells by regulating mitotic cloning and PPAR PPARγ [25]. Yang et al. [23] exposed 3T3-L1 cells to 0–20 μM BDE-47 and found that BDE-47 can upregulate purine metabolism, alter glutathione metabolism, promote adipocyte oxidative stress and uric acid production, and induce adipocyte differentiation by providing a favourable oxidative environment. The advantage of transcriptome sequencing is that it can detect the mRNA expression of multiple genes at the same time. In previous studies, several genes related to a certain pathway were determined. The hub genes were calculated and then the hub genes related transcription factors predicted. Multiple pathways screened out through enrichment analysis, which provided a broader idea for further studying the effect of BDE-47 on adipocyte differentiation.

An interaction network was established between transcription factor genes and hub genes. Pparg is a member of the nuclear receptor transcription factor superfamily that regulates the expression of target genes. Studies have shown that phosphorylation of PPARγ by mitogen-activated protein kinase leads to inhibition of the transcriptional activation function of PPARγ ligands. The transcriptional activation of adipocytes can be regulated by the signal transduction pathway of cytokines involved in the process of adipocyte differentiation [28]. As early as 2013, Bastors et al. [24] proposed PPARγ and related genes as targets of BDE-47. The study of Yang et al. [23] indicated that the expression of PPARγ was significantly increased by 10 μM BDE-47, but it is not clear whether BDE-47 functions through PPARγ. Since Pparg itself is a transcription factor gene, its related transcription factors cannot be directly queried in the NetworkAnaylst database, therefore it was queried whether there were any common transcription factors related to the 9 hub genes in the PPARγ pathway, and *KAT2A*, *MAX*, *SIN3A*, *TBP*, and *EP300* were identified as common transcription factor genes. The common hub genes in the PPARγ pathway included *Aurkb*, *Myc*, *Actb*, and *Aurka*. The *KAT2A* is involved in a wide range of biological events, such as gene regulation, cell proliferation, metabolism, and inflammation [29] and *Myc* protein can stimulate cell cycle progression, inhibit differentiation, and prevent cells from entering a static state. One study detected a specific *Myc*/*MAX* complex on day 1 of 3T3-L1 cell differentiation, which disappeared at subsequent time points. Max is present throughout adipocyte differentiation, while *Myc* is expressed during and after the explosion of 3T3-L1 cell differentiation and proliferation [30]. The main function of Sin3A during 3T3-L1 cell differentiation is mediated through the *SIN3A*/*HDAC* transcription corepressor complex, which interacts with various transcription factors, and directly or indirectly regulates a variety of cellular functions and maintains cell homoeostasis [31]. In vitro studies have confirmed that TBP interacts with PPARγ to form a complex and TBP, as a cofactor of PPARγ, is an integral part of the core transcription mechanism that plays an indispensable role in adipocyte gene expression [32]. During 3T3-L1 cell differentiation, *EP300* directly binds to PPARγ2 as an activator to regulate the expression of its target genes [33]. The *Aurkb*, *Myc*, *Actb*, and *Aurka* genes were closely associated with the regulation of the cell cycle and mitosis. These findings suggest that PPARγ may promote adipocyte differentiation and lipid accumulation through regulation of *KAT2A*, *MAX*, *SIN3A*, *TBP*, *EP300*, *Aurkb*, *Myc*, *Actb*, and *Aurka*.Taken together, this data suggests that BDE-47 May regulate PPARγ and mitosis to influence adipocyte differentiation. We speculate that BDE-47 May regulate adipocyte differentiation by affecting PPARγ and mitosis. However, further research is needed to elucidate the exact mechanisms of these interactions.

Finally, a ‘U’ distribution between PPARγ mRNA expression and BDE-47 concentration was observed, which was like the relationship between the degree of adipocyte differentiation and BDE-47 dose, so it was speculated that the relationship between BDE-47 and adipocyte differentiation may be different from the concentration dependent adipogenic effect in previous studies, but the specific mechanism of action requires further study. This research was limited by the fact that the effects of BDE-47 exposure were only examined on day 8 of 3T3-L1 differentiation, so this data only revealed the overall effect of BDE-47 on 3T3-L1 cell differentiation, and did not examine the effect of BDE-47 exposure throughout the various stages of adipocyte differentiation. In addition, we proposed some action model of BDE-47 based on transcriptome data analysis, but we lack further experiments to verify it.

## 4. Materials & methods

### 4.1. Materials

Biological safety cabinet (Model KS-12, Thermo Fisher Scientific, USA),CO2 incubator (Galaxy 17OR, Thermo Fisher Scientific, USA),NanoDrop 2000 spectrophotometer for nucleic acid and protein analysis (Thermo Fisher Scientific, USA),Benchtop high-speed refrigerated centrifuge (Model 5415 R, Eppendorf, Germany)

Real-Time PCR System (QuantStudio TM 6 Flex Real-Time PCR System, Applied Biosystems, USA),3T3-L1 cell line (Shanghai Institutes for Biological Sciences, Chinese Academy of Sciences),DMEM high glucose culture medium, newborn calf serum, foetal bovine serum, penicillin/streptomycin solution, trypsin, phosphate-buffered saline (PBS) (all purchased from Hyclone, USA),BDE-47 (≥98%, Shanghai Yuanye Bio-Technology Co., Ltd., China),2,4-thiazolidinedione (TZD, Shanghai Macklin Biochemical Co., Ltd., China), MTT powder (MT Company, USA),Oil Red O staining solution, dimethyl sulphoxide (DMSO) (both purchased from Sigma-Aldrich, USA),

3-isobutyl-1-methylxanthine (IBMX), dexamethasone (DEX), insulin (all purchased from Solarbio Science & Technology Co., Ltd., Beijing, China),Reverse Transcription Kit (Thermo Fisher Scientific, USA),RT-PCR Kit (TaKaRa, Japan).

### 4.2. Methods

#### 4.2.1. Induced differentiation of 3T3-L1 cells

**Figure 8.**
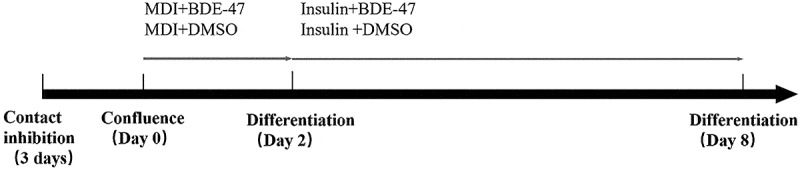
Induction and differentiation of 3T3-L1 cells exposed to BDE-47.

For this study, we conducted experiments with three biological replicates per treatment group to ensure statistical robustness. The 3T3-L1 preadipocytes were cultured as described previously [34]. Briefly, 3T3-L1 preadipocytes were seeded into 6-well plates at a density of 1 × 105 and maintained in fresh DMEM high glucose complete culture medium containing 10% newborn calf serum and 1% penicillin/streptomycin in an atmosphere of 5% CO2 and at 37°C for two days until the cells became confluent. Differentiation was induced by treating the cells with 0.5 mmol/L methylisobutylxanthine, 1 µmol/L dexamethasone, and 1.67 µmol/L insulin DMEM high glucose complete medium on the third day of culture or day 0 of differentiation [22]. Cells were also exposed to either 0.1% DMSO as a control group or drug treatment groups containing different concentrations of BDE-47 at 2.5, 7.5, 12.5, 18.75, and 25 µmol/L. In our experiments, each treatment was applied to three biological replicates to ensure statistical robustness and reproducibility of the results.On day 2 of differentiation, the culture medium was replaced with DMEM high glucose medium containing 1.67 µmol/L insulin, fresh BDE-47 and DMSO was also added, and the solution was changed every two days until the end of the experiment at day 8 of differentiation. The composition of each BDE-47 treatment group is shown in Table 1, and the specific procedure of cell differentiation and drug treatment is outlined in Figure 8. In our study, we used a one-way analysis of variance (ANOVA) followed by Tukey’s post-hoc test to compare the means of multiple groups. we conducted experiments with three biological replicates per treatment group to ensure statistical robustness.

**Table 1.** Preparation of BDE-47 treatment groups.

Group	BDE-47 (μM)	1 mm BDE-47 (μl)	DMEM (μl)	Total (μl)
BDE_47JH	25	50	1950	2000
BDE_47H	18.75	37.5	1962.5	2000
BDE_47M	12.5	25	1975	2000
BDE_47L	7.5	15	1985	2000
BDE_47JL	2.5	5	1995	2000

#### 4.2.2. Oil red O staining

The formation of lipid droplets was observed by oil red O staining on day 8 of 3T3-L1 cell differentiation [35]. The original culture medium was discarded and cells were washed twice with pre-cooled sterile PBS. Cells were then fixed in 4% paraformaldehyde at room temperature for 60 min, followed by staining with 1.5 mL oil red O diluent (0.5% oil red O solution and deionized water at a ratio of 3:1) for 30 to 60 min. Samples were washed three times with pre-cooled sterile PBS to remove excess dye. Next, samples were incubated with 60% isopropanol for 30 min at room temperature. The solution was transferred to 96-well plates, and quantitative analysis of the oil red O staining was performed by measuring the absorbance value of each well at 510 nm in a microplate reader. For quantitative data that adhered to a normal distribution, we described the results using the mean ± standard deviation (SD). Comparisons between multiple groups were conducted using one-way ANOVA to determine the overall statistical significance. Subsequent pairwise comparisons between groups were performed using the Tukey post-hoc test. A p-value of less than 0.05 was considered statistically significant.

#### 4.2.3. RNA-seq

The extraction and quality inspection of total RNA, construction of the library and computer sequencing, data filtering and quality control and comparative bioinformatics analysis of reference genome were conducted by Beijing Nuohe Zhiyuan Technology Co., Ltd. (China) [36]. After strict data filtering and quality control, the corresponding analysis results were obtained. A corrected P-value of less than 0.05 was statistically significant.

#### 4.2.4. Identification of DEGs and common genes

The R 4.0.1 limma package was used to identify differentially expressed genes (DEGs) between the BDE-47-exposed groups and control group [37]. Cut-off criteria were obtained using a P-value <0.05. Identification of common genes between the DEGs of each BDE-47 treatment and control group was obtained using the R 4.0.1 limma package.

#### 4.2.5. Short time-series expression Miner

STEM (Short Time-series Expression Miner v1.3.13) [38] software is suitable to analyse the time-axis in the transcriptome sequencing. It can cluster the sequencing data grouped by time or dose, and analyse the characteristics of its expression pattern, and look for genes that change regularly with the change of exposure concentration.

#### 4.2.6. Identification of hub genes

Hub genes were identified using the Cytoscape plug in cytoHubba (http://apps. cytoscape.org/apps/cytohubba)[38]. CytoHubba contains 11 topology analysis methods, and uses the topology algorithm to analyse the individual hub genes.

#### 4.2.7. Gene set enrichment analysis

The Gene Ontology (GO), Kyoto Encyclopedia of Genes and Genomes (KEGG), Reactome, and Wikipathways databases were used to enrich and analyse hub genes. The GO term was organized in three categories as biological process, molecular function, and cellular component [39]. The KEGG pathway was used to identify metabolic pathways [39]. For the purpose of significant pathway analysis, Wikipathways [40] and Reactome [41] databases were also used.Gene set enrichment analysis was performed using GSEA software provided by the Broad Institute.

#### 4.2.8. Transcription factor genes interactions

The NetworkAnalyst (https://www.networkanalyst.ca/)[42] platform was used to establish the interaction network between transcription factor genes and hub genes. NetworkAnalyst is a comprehensive database for performing gene expression analysis for numerous species. Searching for specific genes and transcription factors in signal transduction pathway by GeneCards database (https://www.genecards.org) [43].

#### 4.2.9. Real-time polymerase chain reaction (qPCR)

PPARγ gene was selected to verify the transcriptome sequencing results. On day 8 of differentiation in each treatment group, 1 mL of precooled Trizol was added to each well to extract total RNA, and cDNA synthesis was performed according to the instructions of the reverse transcription kit (Thermo Fisher Scientific, USA). The reaction conditions used were incubation at 65°C for 5 min, cooling on ice, 2000 × g centrifugation for 30 s, incubation at 42°C for 60 min, and termination of the reaction after incubation at 70°C for 5 min. The RT-PCR reaction conditions were pre-denaturation at 94°C for 30 s, then denaturation at 94°C for 5 s and annealing at 60°C for 30 s for 40 cycles. The Primer 5 program was used to design the primer sequences (Table 2). All primers were synthesized by Shanghai Bioengineering Co., Ltd. (China). β-actin was used as an internal reference gene. The relative expression of PCR products was analysed by 2^−ΔΔCt^.

**Table 3.** Hub gene topological analysis scores.

Node name	MCC	DMNC	MNC	Degree	EPC	BottleNeck	EcCentricity	Closeness	Radiality	Betweenness	Stress	Clustering Coefficient
Actb	4.55E + 12	0.23868	121	123	57.051	44	0.19812	346.6167	7.823	65682.15	818468	0.11049
Cdk1	9.22E + 13	0.76258	82	83	64.183	2	0.1651	304.4667	7.52362	9987.989	162808	0.4017
Myc	1.68E + 12	0.35383	75	78	51.884	25	0.19812	315.7667	7.66469	22250.47	343986	0.18149
Ccnb1	9.22E + 13	0.86414	74	74	62.934	5	0.1651	299.1	7.50011	7083.442	131076	0.48167
Aurkb	9.22E + 13	0.88307	72	74	63.069	5	0.1651	297.7167	7.48287	7440.551	132376	0.46983
Plk1	9.22E + 13	0.84765	73	73	61.89	9	0.1651	294.9167	7.45466	4622.721	105518	0.47451
Aurka	9.22E + 13	0.89007	71	71	61.889	1	0.1651	295.3833	7.46563	5498.665	118912	0.50262
Pparg	2655431	0.26381	68	69	31.582	17	0.19812	300.55	7.53459	25276.52	275914	0.14663
Kif11	9.22E + 13	0.94098	68	68	60.36	1	0.1651	286.1167	7.37942	3189.296	67346	0.53863
Casp3	6.10E + 08	0.3429	66	68	43.668	7	0.19812	304.0667	7.58005	13175.8	225676	0.18657

**Table 4.** Gene set enrichment analysis of GO terms.

Category	GO ID	GO pathway	P-value	Genes
Biological Process	GO:0051726	regulation of cell cycle	1.64E-07	Kif11,Cdk1,Aurkb,Myc,Aurka,Plk1,Ccnb1,Casp3,Actb
	GO:1902850	microtubule cytoskeleton organization involved in mitosis	6.09E-06	Kif11,Aurkb,Aurka,Plk1,Ccnb1
	GO:0140014	mitotic nuclear division	1.15E-05	Kif11,Aurkb,Aurka,Plk1,Ccnb1
	GO:0051098	regulation of binding	2.26E-05	Pparg,Aurkb,Myc,Aurka,Plk1,Actb
	GO:0022402	cell cycle process	2.37E-05	Kif11,Cdk1,Aurkb,Myc,Aurka,Plk1,Ccnb1
	GO:0018105	peptidyl-serine phosphorylation	2.46E-05	Cdk1,Aurkb,Aurka,Plk1,Ccnb1
	GO:1903047	mitotic cell cycle process	3.41E-05	Kif11,Cdk1,Aurkb,Aurka,Plk1,Ccnb1
	GO:0051301	cell division	3.97E-05	Kif11,Cdk1,Aurkb,Aurka,Plk1,Ccnb1
	GO:1901857	positive regulation of cellular respiration	3.97E-05	Cdk1,Myc,Ccnb1
	GO:1903862	positive regulation of oxidative phosphorylation	3.97E-05	Cdk1,Myc,Ccnb1
Molecular Function	GO:0035173	histone kinase activity	6.63E-07	Cdk1,Aurkb,Aurka,Ccnb1
	GO:0019899	enzyme binding	3.22E-05	Pparg,Kif11,Aurkb,Myc,Aurka,Plk1,Ccnb1,Casp3,Actb
	GO:0019900	kinase binding	0.00071	Kif11,Aurkb,Aurka,Plk1,Ccnb1,Actb
	GO:0004672	protein kinase activity	0.0034	Cdk1,Aurkb,Aurka,Plk1,Ccnb1
	GO:0035174	histone serine kinase activity	0.0054	Aurkb,Aurka
	GO:0005515	protein binding	0.0055	Pparg,Kif11,Cdk1,Aurkb,Myc,Aurka,Plk1,Ccnb1,Casp3,Actb
	GO:0019901	protein kinase binding	0.0055	Kif11,Aurka,Plk1,Ccnb1,Actb
	GO:0005524	ATP binding	0.0057	Kif11,Cdk1,Aurkb,Aurka,Plk1,Actb
	GO:0036094	small molecule binding	0.0066	Pparg,Kif11,Cdk1,Aurkb,Aurka,Plk1,Actb
	GO:0004674	protein serine/threonine kinase activity	0.01	Cdk1,Aurkb,Aurka,Plk1
Cellular Component	GO:0005819	spindle	5.90E-08	Kif11,Cdk1,Aurkb,Myc,Aurka,Plk1,Ccnb1
	GO:0005876	spindle microtubule	5.90E-08	Kif11,Cdk1,Aurkb,Aurka,Plk1
	GO:0000780	condensed nuclear chromosome, centromeric region	1.02E-06	Aurkb,Aurka,Plk1,Ccnb1
	GO:0000922	spindle pole	3.52E-06	Kif11,Aurkb,Aurka,Plk1,Ccnb1
	GO:0005856	cytoskeleton	5.30E-05	Kif11,Cdk1,Aurkb,Myc,Aurka,Plk1,Ccnb1,Actb
	GO:0072686	mitotic spindle	5.30E-05	Kif11,Cdk1,Aurkb,Aurka
	GO:0099080	supramolecular complex	5.30E-05	Kif11,Cdk1,Aurkb,Aurka,Plk1,Ccnb1,Actb
	GO:0099513	polymeric cytoskeletal fibre	5.30E-05	Kif11,Cdk1,Aurkb,Aurka,Plk1,Actb
	GO:0032991	protein-containing complex	6.01E-05	Pparg,Kif11,Cdk1,Aurkb,Myc,Aurka,Plk1,Ccnb1,Casp3,Actb
	GO:0051233	spindle midzone	0.00012	Aurkb,Aurka,Plk1

**Table 5.** Top pathways identified using the KEGG, reactome, and WikiPathways databases.

Database	Term ID	Pathways	P-value	Genes
KEGG	mmu04914	Progesterone-mediated oocyte maturation	2.89E-05	Cdk1,Aurka,Plk1,Ccnb1
	mmu04110	Cell cycle	3.74E-05	Cdk1,Myc,Plk1,Ccnb1
	mmu04114	Oocyte meiosis	3.74E-05	Cdk1,Aurka,Plk1,Ccnb1
	mmu04115	p53 signalling pathway	0.00049	Cdk1,Ccnb1,Casp3
	mmu04218	Cellular senescence	0.0047	Cdk1,Myc,Ccnb1
	mmu05205	Proteoglycans in cancer	0.0066	Myc,Casp3,Actb
	mmu05132	Salmonella infection	0.0069	Myc,Casp3,Actb
	mmu05170	Human immunodeficiency virus 1 infection	0.0069	Cdk1,Ccnb1,Casp3
	mmu05216	Thyroid cancer	0.0069	Pparg,Myc
	mmu05416	Viral myocarditis	0.0235	Casp3,Actb
Reactome	MMU-174143	APC/C-mediated degradation of cell cycle proteins	4.11E-06	Cdk1,Aurkb,Aurka,Plk1,Ccnb1
	MMU-2980767	Activation of NIMA Kinases NEK9, NEK6, NEK7	2.17E-05	Cdk1,Plk1,Ccnb1
	MMU-176417	Phosphorylation of Emi1	2.44E-05	Cdk1,Plk1,Ccnb1
	MMU-6804114	TP53 Regulates Transcription of Genes Involved in G2 Cell Cycle Arrest	2.79E-05	Cdk1,Aurka,Ccnb1
	MMU-2565942	Regulation of PLK1 Activity at G2/M Transition	0.00015	Cdk1,Aurka,Plk1,Ccnb1
	MMU-74160	Gene expression (Transcription)	0.00015	Pparg,Cdk1,Aurkb,Myc,Aurka,Ccnb1,Actb
	MMU-176412	Phosphorylation of the APC/C	0.00026	Cdk1,Plk1,Ccnb1
	MMU-2500257	Resolution of Sister Chromatid Cohesion	0.00038	Cdk1,Aurkb,Plk1,Ccnb1
	MMU-69273	Cyclin A/B1/B2 associated events during G2/M transition	0.00038	Cdk1,Plk1,Ccnb1
	MMU-212436	Generic Transcription Pathway	0.0008	Pparg,Cdk1,Aurkb,Myc,Aurka,Ccnb1
WikiPathways	WP2902	p53 signalling	0.0063	Cdk1,Ccnb1,Casp3
	WP2432	Spinal cord injury	0.0098	Cdk1,Myc,Casp3
	WP116	Hedgehog signalling pathway	0.0271	Cdk1,Ccnb1

**Table 2.** Primer sequences used for real-time PCR reactions.

Genes	Sequence	Length
β-actin	F:AGAGGGAAATCGTGCGTGACATCAAAGAG	209bp
	R:GATGCCACAGGATTCCATACCCAAGAAGG	
PPARγ	F:CGCCAAGGTGCTCCAGAAGATC	104bp
	R:GGTGAAGGCTCATGTCTGTCTCTG	

## 5. Conclusions

The 3T3-L1 cells were exposed to different doses of BDE-47 to examine the effect of BDE-47 on 3T3-L1 cell differentiation and explore its mechanism of action. The results suggest that BDE-47 May promote 3T3-L1 cellular differentiation and induce 3T3-L1 lipid accumulation through regulation of the PPARγ and mitotic pathways. A bimodal distribution was observed between BDE-47 concentration and both PPARγ mRNA expression and adipocyte differentiation.

## Data Availability

The data that support the findings of this study are openly available in figshare at https://doi.org/10.6084/m9.figshare.27013795.v4, reference number 27,013,795.
